# Enhancement of Mucosal Immunogenicity of Viral Vectored Vaccines by the NKT Cell Agonist Alpha-Galactosylceramide as Adjuvant

**DOI:** 10.3390/vaccines2040686

**Published:** 2014-10-10

**Authors:** Shailbala Singh, Pramod N. Nehete, Guojun Yang, Hong He, Bharti Nehete, Patrick W. Hanley, Michael A. Barry, K. Jagannadha Sastry

**Affiliations:** 1 Department of Immunology, The University of Texas M.D. Anderson Cancer Center, Houston, TX 77030, USA; E-Mails: ssingh1@mdanderson.org (S.S.); gyang3@mdanderson.org (G.Y.); 2 Department of Veterinary Sciences, The University of Texas M.D. Anderson Cancer Center, Bastrop, TX 78602, USA; E-Mails: pnehete@mdanderson.org (P.N.N.); bpnehete@mdanderson.org (B.N.); 3 Department of Stem Cell Transplantation and Cellular Therapy, The University of Texas M.D. Anderson Cancer Center, Houston, TX 77030, USA; E-Mail: hhe@mdanderson.org; 4 Rocky Mountain Veterinary Branch, Division of Intramural Research, National Institute of Allergy and Infectious Diseases, National Institutes of Health Rocky Mountain Laboratories, Hamilton, MT 59840, USA; E-Mail: patrick.hanley@nih.gov; 5 Department of Internal Medicine, Division of Infectious Diseases, Mayo Clinic, Rochester, MN 55902, USA; E-Mail: Barry.Michael@mayo.edu; 6 Department of Molecular Medicine, Mayo Clinic, Rochester, MN 55902, USA; 7 Department of Immunology, Mayo Clinic, Rochester, MN 55902, USA; 8 Translational Immunovirology and Biodefense Program, Mayo Clinic, Rochester, MN 55902, USA

**Keywords:** adenovirus vector, vesicular stomatitis viral vector, intranasal, HIV vaccine, Rhesus macaques, alpha-galactosylceramide, mucosal immunity, NKT cells

## Abstract

Gene-based vaccination strategies, specifically viral vectors encoding vaccine immunogens are effective at priming strong immune responses. Mucosal routes offer practical advantages for vaccination by ease of needle-free administration, and immunogen delivery at readily accessible oral/nasal sites to efficiently induce immunity at distant gut and genital tissues. However, since mucosal tissues are inherently tolerant for induction of immune responses, incorporation of adjuvants for optimal mucosal vaccination strategies is important. We report here the effectiveness of alpha-galactosylceramide (α-GalCer), a synthetic glycolipid agonist of natural killer T (NKT) cells, as an adjuvant for enhancing immunogenicity of vaccine antigens delivered using viral vectors by mucosal routes in murine and nonhuman primate models. Significant improvement in adaptive immune responses in systemic and mucosal tissues was observed by including α-GalCer adjuvant for intranasal immunization of mice with vesicular stomatitis virus vector encoding the model antigen ovalbumin and adenoviral vectors expressing HIV env and Gag antigens. Activation of NKT cells in systemic and mucosal tissues along with significant increases in adaptive immune responses were observed in rhesus macaques immunized by intranasal and sublingual routes with protein or adenovirus vectored antigens when combined with α-GalCer adjuvant. These results support the utility of α-GalCer adjuvant for enhancing immunogenicity of mucosal vaccines delivered using viral vectors.

## 1. Introduction 

Among the different strategies for delivering vaccine antigens, viral vectors merit attention because of their potency in reaching wider cell types and efficient antigen expression achieving sustained levels of antigen-specific immune responses [1]. In particular, adenoviral (Ad) vectors have been extensively investigated because of their obvious potency in reaching multiple cell types and robust levels of *in vivo* antigen expression [2]. Commonly employed Ad vectors include replication defective strains engineered to eliminate most of the adenoviral antigens allowing expression and immunogenicity of the transgene. However, Ad serotype 5 (Ad5) based HIV vaccines tested in the past few years proven ineffective, specifically in individuals with pre-existing Ad5 immunity [3,4,5,6,7,8]. To overcome this concern, we tested serotype-switching strategy employing other serotypes, Ad1, 2 and 6 that proved significantly more immunogenic than multiple doses of Ad5 vaccine and also afforded relatively better control of viremia after pathogenic virus challenge [9,10,11]. 

Since mucosal tissues constitute the major portals of HIV-1 entry worldwide and barrier protective immunity at these sites is important, we used the Ad serotype switching strategy to test protective efficacy of HIV-1 vaccine immunogen delivered by the mucosal intra-vaginal route in comparison to the systemic intramuscular immunization in the rhesus macaque model [11]. We observed that intramuscular immunization generated stronger systemic cellular immune responses than the intra-vaginal route, but the latter yielded higher mucosal immunity, specifically antigen-specific central memory T cells (T_cm_) subset along with more animals in this group exhibiting lower viral loads [11]. 

Since mucosal surfaces are inherently resistant to immunity, addition of adjuvants to the vaccine formulations is often essential for optimal generation of adaptive immunity at these sites [12,13,14]. While bacterial toxins, both wild type and mutated versions, have proven to be strong mucosal adjuvants, potential safety concerns preclude clinical utility [15,16]. We reported earlier the effectiveness of alpha-galactosylceramide (α-GalCer), a synthetic glycolipid to function as an adjuvant for peptide and protein antigens delivered by the oral and nasal routes [17,18,19]. Because α-GalCer is a potent agonistic ligand for natural killer T (NKT) cells, its use in vaccination strategies allows bridging of the innate and adaptive arms of the immune system resulting in broadly disseminated antigen-specific immunity [20,21]. Here we report the effectiveness of α-GalCer as adjuvant for enhancing mucosal immunogenicity of viral vectored, specifically recombinant Ad vector-based antigens in mice and nonhuman primate models. In both mice and rhesus macaques, mucosal immunization with viral vectored antigens in the presence of α-GalCer significantly increased systemic as well as antibody and T cell immune responses. 

## 2. Experimental 

### 2.1. Animals 

Female Balb/C and C57BL/6 mice aged 6–10 weeks were purchased from the National Cancer Institute (Frederick, MD, USA). The animals were maintained in a specific pathogen-free environment at the institutional animal facility. Adult female rhesus macaques (*Macaca mulatta*) of Indian origin maintained in the specific pathogen-free breeding colony at the Michael Keeling Center for Comparative Medicine and Research of The University of Texas MD Anderson Cancer Center, Bastrop TX, were used in the studies. All study rhesus monkeys are maintained in accordance with the “Guide for the Care and Use of Laboratory Animals” of the Institute of Laboratory Animal Resources; National Research Council. The facility is fully accredited by the Association for the Assessment and Accreditation of Laboratory Animal Care International. All animal procedures were conducted in compliance with the institutionally approved protocols.

### 2.2. Cell Lines and Cell Cultures

Murine T lymphoma (thymoma) cell line EL-4 (C57BL/6, H-2b) and murine mastocytoma cell line P815 (Balb/C, H-2d) were maintained in RPMI 1640 (Thermo Scientific Hyclone, Logan, UT, USA), supplemented with 10% heat inactivated fetal bovine serum (FBS) (Atlanta Biologicals, Lawrenceville, GA, USA), 50 U/mL of penicillin-streptomycin (Thermo Scientific Hyclone, Logan, UT, USA) and 50 μg/mL gentamycin (Lonza Biowittaker, Walkersville, MD, USA). 

### 2.3. Reagents

The synthetic peptides corresponding to the H-2^b^-restricted cytotoxic T lymphocyte (CTL) epitope (SIINFEKL) and I-A^b^-restricted CD4^+^ T helper (T_h_) lymphocyte epitope (ISQAVHAAHAEINEAGR) of chicken ovalbumin were purchased from Peptides International Inc. (Louisville, KY, USA), and dissolved in 1× PBS at a concentration of 2.5 mg/mL. The HIV-1 envelope and gag peptides specific to H-2d-restricted CTL epitopes, RKRIHIGPGRAFYTT and VGGHQAAMQMLKDTINEEAA respectively and I-Ad-restricted CD4 T_h_ epitopes TVQCTHGIRPVVSTQ and TSNPPIPVGDIYKRWIILGL were obtained from the AIDS Research and Reference Reagent Program (Germantown, MD, USA). The alpha-galactosylceramide (α-GalCer) was purchased from Diagnocine LLC (Hackensack, NJ, USA) and dissolved in dimethyl sulfoxide, (Sigma, St. Louis, MO, USA) at a concentration of 1 mg/mL. Recombinant Influenza A, nucleocapsid protein (NP) was obtained from IMGENEX (San Diego, CA, USA). Vesicular stomatitis virus expressing chicken ovalbumin (VSV-OVA) was kindly provided by Dr. Kimberly Schluns (The UT MD Anderson Cancer Center, Houston, TX, USA). 

### 2.4. Immunizations in Mice

For intranasal immunization, mice were anesthetized by intraperitoneal (i.p.) injection of ketamine and xylazine hydrochloride (100 mg/kg and 10 mg/kg respectively). Replication defective first generation adenoviral vectors with deletion of E1 gene (FG-Ad) expressing HIV-1 envelope (FG-Ad-env) and HIV-1 gag genes (FG-Ad-Gag) [9] were administered to Balb/C mice at a dose of 5 × 10^9^ viral particles (vp) either alone or with 2 µg of α-GalCer in the nose using the previously described procedure [17]. Similarly, vesicular stomatitis virus expressing ovalbumin (VSA-OVA) was administered at a dose of 2.5 × 10^4^ plaque forming units (pfu) intranasally to C57BL/6 mice either alone or with 2 µg of α-GalCer. All mice received a single immunization and the adaptive immune responses in different tissues were determined at day 14 post-immunization. 

### 2.5. Immunization and Sample Collection in Rhesus Macaques

Monkeys in the various experiments were immunized by intranasal, sublingual, intravenous, or intra-muscular route and where the α-GalCer adjuvant was used the concentration was 125 µg/animal (1 mg/mL stock prepared in dimethyl sulfoxide, DMSO). Animals either received 20 µg of recombinant influenza virus nucleocapsid (NP) protein or 10^11^ viral particles (vp) of helper dependent adenovirus vector (HD-Ad) expressing HIV-1 envelope [9]. The animals were anesthetized during all procedures to minimize discomfort. 

For all mucosal immunizations, the animals were anesthetized as described previously [9,11] and 100 µL of the inoculum was administered by either intranasal or sublingual route. The animals were maintained in the lateral recumbent position for at least 3–5 min to avoid leakage of the inoculum either out of the nostril or swallowing. Similarly for systemic, intravenous, and intramuscular immunizations, anesthetized animals were administered 100 µL of the inoculum in the cephalic vein and quadriceps muscles, respectively. Blood samples, bronchial alveolar lavage (BAL) and biopsies from liver, lymph nodes, colon, and vagina were collected at different time points as described previously to analyze the activation of NKT cells and antigen specific adaptive immune responses [9,11].

### 2.6. IFN-γ ELISpot Assay

Antigen-specific responses of cells isolated from cervical lymph nodes, mediastinal lymph nodes (MdLN), iliac lymph nodes, lungs, and spleens of the immunized mice were determined by IFN-γ ELISpot assay as described previously [17,19]. The cells were stimulated by incubating with medium, Concavalin A (5 µg/mL), ovalbumin or HIV-1 envelope or gag specific peptides representing CD4 and CD8 epitopes (1 µM) for 48 h before secondary antibody treatment and color development to detect IFN-γ spot forming cells (SFC) using the commercial reagent kit (BD Biosciences, San Jose, CA, USA). For rhesus macaques, single cell suspensions from blood or tissues collected as above were stimulated with the mitogens Con A and the NP protein or the HIV envelope peptide pools (HIV-1_JR-FL_, AIDS Research and Reference Reagent Program, Germantown, MD, USA) to determine the numbers of IFN-γ-producing cells by the ELISpot assay (Millipore, Bedford, MA, USA) using the methodology reported earlier [22,23]. Enumeration of spots representing individual cells producing IFN-γ was done by Zellnet Consulting Inc., Fort Lee NJ using KS-ELISPOT automatic system (Carl Zeiss Inc., Thornwood, NY, USA). Responses were considered positive only when they were above 10 SFC/2 × 10^5^ input cells for mice and 5 SFC/1 × 10^5^ input cells for rhesus macaques and at least twice the number obtained in cells cultured with medium alone. 

### 2.7. Analyses of Antigen Specific CTL Responses

The antigen-specific cytotoxic T lymphocyte (CTL) response of cells isolated from the spleens of immunized mice was determined by a previously described ^51^Cr Release Assay [17]. Splenocytes were re-stimulated *in vitro* for 5 days with OVA peptide (SIINFEKL) or HIV envelope peptide (RKRIHIGPGRAFYTT) before assaying for cytolytic activity by co-culturing with ^51^Cr-labeled syngeneic EL-4 or P815 target cells treated with either the cognate peptide or culture medium. The percentage (%) of specific lysis was calculated using the following formula: % specific lysis = (experimental release − spontaneous release)/(maximum release − spontaneous release) × 100, where the spontaneous release represents the radioactivity obtained when the target cells were incubated in culture medium without effectors and maximum release represents the radioactivity obtained when the target cells were lysed with 5% Triton X-100.

### 2.8. Enumeration of Antigen-Specific CD8 T Lymphocytes

Presence of antigen-specific CD8^+^ T cells prior to, and after, boosting immunization was determined using H^2b^ tetramer complexed with the OVA CD8^+^ T cell epitope peptide (SIINFEKL). Briefly, cells were stained with allophycocyanin (APC)-conjugated major histocompatibility complex (MHC)-I tetramer complexed with OVA peptide (provided by Leo Lefrancois, University of Connecticut, Storrs, CT, USA), PE-conjugated anti-CD44 (clone IM7 BD Biosciences, San Jose, CA, USA), PerCP Cy5.5 conjugated anti-CD8 (clone 53–6.7 BD Biosciences, San Jose, CA, USA) and FITC-conjugated anti-CD62L (clone MEL-14 BD Biosciences, San Jose, CA, USA) antibodies. Cells were also stained with Aqua Live/Dead reagent (Invitrogen, Carlsbad, CA, USA) to select live cells for all analyses. Percentage of OVA-tetramer positive cells within CD44^hi^ and CD8^+^ live lymphocytes was determined for animals receiving immunization with either OVA alone or OVA + α-GalCer.

### 2.9. Antigen Specific Antibody Response

Antigen specific antibody responses were evaluated in the blood and vaginal washes of immunized mice. Blood samples were collected from the retro-orbital sinuses. Vaginal washes were collected by repeated flushing with PBS. Serum (dilution 1:200) and mucosal secretions (dilution 1:10) were assayed for antibody levels to OVA or HIV gp 120 or HIV Gag by ELISA using standard protocols [24]. Horseradish peroxidase (HRP)-conjugated goat antibodies to mouse IgG or IgA (KPL Inc., Gaithersburg, MD, USA) were used for detection. For each group of immunized mice, results were expressed as geometric mean titer (GMT) ± SD. Monkey serum (dilution 1:10) and BAL and vaginal wash (dilution 1:2) IgG and IgA responses specifically against NP and HIV-1 envelope protein were determined by a standard ELISA. Biotinylated anti-human IgG and anti-human IgA (Mabtech Inc., Cincinnati, OH, USA) were used as detection antibodies followed by streptavidin-alkaline phosphatase (KPL Inc., Gaithersburg, MD, USA) to detect the signal after addition of the para-nitrophenylphosphate (PNPP) substrate (Thermo Fisher Scientific Inc., Waltham, MA, USA) 

### 2.10. Cytokine Measurements in Rhesus Macaques 

Concentration of serum cytokines, IL4 and IFN-γ, at different time points after administration of α-GalCer was measured by non-human primate cytokine bead array kit (Millipore Corp., Billerica, MA, USA) according to the manufacture’s protocol [11]. Multianalyte profiling was performed on Bio-Rad 200 system and the analyses of fluorescence data was performed on the Bio-Plex manager 5.0 (Bio-Rad, Hercules, CA, USA).

### 2.11. Flow Cytometry Analyses of Activation of NKT and T Cells

A series of commercially available human monoclonal antibodies that cross reacts with rhesus mononuclear cells were used in flow cytometry analyses as described previously [11,25]. Cell-surface markers were determined using the following fluorescence labeled monoclonal antibodies specific to different lymphocytes subsets: CD3 (FITC, clone SP34-2, BD Pharmingen, San Jose, CA, USA), CD4 (PE, clone L200, BD Pharmingen), CD8 (PE, clone RPT, Invitrogen, BD Pharmingen) and APC-conjugated human CD1d tetramer loaded with PBS57 (provided by NIH tetramer facility at Emory University, Atlanta, GA, USA). Live cells were selected using the live-dead fixable dead cells stain kit obtained from Invitrogen (Carlsbad, CA, USA). The CD3^+^ cells from lymphocytes that were gated on forward scatter versus side scatter dot plot were used to analyze for CD1d tetramer^+^ lymphocyte population. Both compensation and fluorescence minus one (FMO) controls were utilized to establish the gating strategy for identifying the NKT population. Results were acquired on an LSRII flow cytometer (BD Biosciences, San Jose, CA, USA). 

### 2.12. Statistical Analysis 

The immune responses were expressed as averages of three to six mice/group. The significance of the difference between different immunization groups was evaluated by two-way ANOVA. All analyses were performed using GraphPad Prism, version 6 (GraphPad Software, San Diego, CA, USA) and *p* ≤ 0.05 was considered statistically significant.

## 3. Results and Discussion 

### 3.1. Enhancement of Systemic and Mucosal Antigen-Specific Immune Responses by α-GalCer after Intranasal Vaccination with Viral Vectored Antigens in Mice 

**Figure 1:**
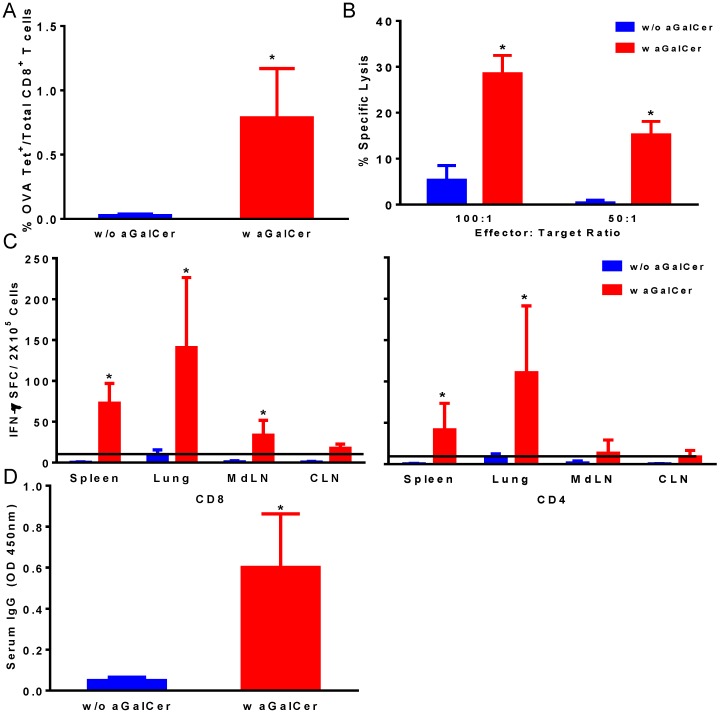
Induction of antigen-specific immune responses by intranasal immunization with vesicular stomatitis viral vector encoding ovalbumin as the model antigen (VSV-OVA) using alpha-galactosylceramide (α-GalCer) adjuvant. Single cell suspensions from spleens were analyzed for the presence of antigen-specific CD8^+^ T lymphocytes by staining with fluorescently labeled CD8 OVA tetramer and antibodies to mouse CD44 and CD8 along with Aqua live/dead stain (**A**). Antigen-specific cytolytic activity of splenocytes isolated from immunized mice was determined by the standard chromium-release assay employing the syngeneic EL-4 target cells pulsed with the OVA peptide, at two different effector to target cell ratios (**B**). Data were adjusted for background by subtracting control values (target cells not pulsed with the OVA peptide). IFN-γ production in response to stimulation with CD8 and CD4 T cell epitope peptides from OVA was determined by using a standard IFN-γ ELISpot assay (**C**). Data are shown as IFN-γ spot forming cells (SFC) per 2 × 10^5^ input cells and OVA specific responses were adjusted to background medium control. Antigen specific IgG antibody responses were determined in serum samples by ELISA (**D**). Data are representative of two separate experiments and expressed as mean ± S.D. Statistical analyses between responses to different immunizations was performed using Mann Whitney Test to determine significant differences (* *p* ≤ 0.05) between immunizations with VSV-OVA alone and VSV-OVA admixed with α-GalCer.

Earlier studies from our group demonstrated the effectiveness of immunizations employing multiple doses of protein antigen admixed with α-GalCer delivered by the mucosal intranasal route to afford repeated activation of NKT cells as well as enhancement of adaptive immune responses [17,19]. In this investigation, we tested the effectiveness of α-GalCer adjuvant for improving immunity induced by antigens expressed from viral vectors employing the vesicular stomatitis viral vector encoding ovalbumin as the model antigen (VSV-OVA). Mice were immunized once by the intranasal route with the VSV-OVA in the presence or absence of the α-GalCer adjuvant and 14 days later analyzed for humoral and cellular immune responses in different systemic and mucosal tissues (Figure 1). We observed significant enhancement in OVA tetramer positive CD8 T cells in the blood of mice that were immunized with the VSV-OVA in the presence of α-GalCer, relative to those with VSV-OVA alone (Figure 1A). This is also reflected in the functional properties of the T cells in terms of significantly higher OVA-specific CTL responses in the spleen (Figure 1B) and IFN-γ producing CD4 and CD8 T cells in spleen and multiple mucosal tissues in mice vaccinated with VSV-OVA + α-GalCer when compared to mice that received VSV-OVA only (Figure 1C). A similar level of effectiveness of the α-GalCer adjuvant was observed for inducing antigen-specific humoral immunity in terms of significantly enhanced production of OVA-specific serum IgG antibodies (Figure 1D).

**Figure 2:**
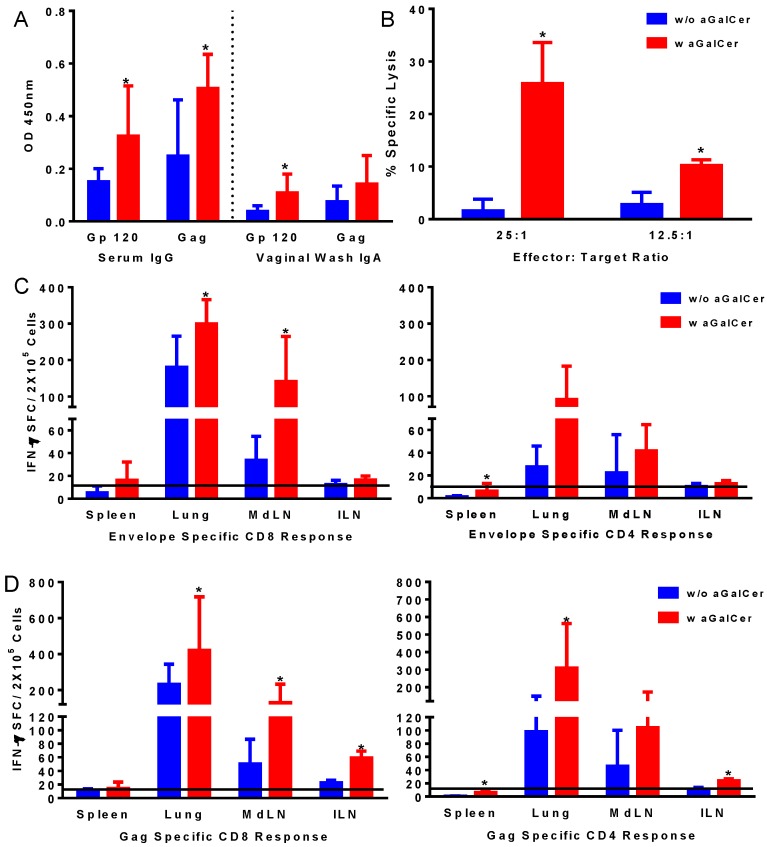
Induction of antigen-specific immune responses by intranasal immunization with adenoviral vectors expressing HIV-env and Gag using α-GalCer adjuvant. Mice were immunized by intranasal route with first generation adenoviral vectors with deletion of E1 gene (FG-Ad) HIV-env and FG-Ad HIV-Gag either alone or admixed with α-GalCer and were sacrificed 14 days later to determine systemic and mucosal adaptive immune responses. Antigen specific IgG and IgA antibody responses to recombinant HIV envelope protein gp120 and HIV Gag (p55) were determined in serum and vaginal washes by enzyme-linked immunosorbent assay (ELISA) (**A**). Antigen-specific cytolytic activity of splenocytes isolated from immunized mice were determined by the standard chromium-release assay employing the syngeneic P815 target cells pulsed with the HIV-gp120 peptide, at different effector to target cell ratios (**B**). Data were adjusted for background by subtracting control values (target cells not pulsed with the peptide). Single cell suspensions from spleen, lungs, mediastinal lymph nodes (MdLN), and Iliac lymph nodes (ILN) were analyzed for interferon-gamma (IFN-γ) production in response to stimulation with CD8 T and CD4 T cell epitope peptides from HIV-env (**C**) and HIV-Gag (**D**) by using a standard IFN-γ ELISpot assay. Data are shown as IFN-γ spot forming cells (SFC) per 2 × 10^5^ input cells and antigen specific responses were adjusted to background medium control. Data are representative of two separate experiments and expressed as mean ± S.D. Statistical analyses between responses to different immunizations were performed using Mann Whitney Test, * (*p* ≤ 0.05) between immunizations with Ad vectors alone and Ad vectors admixed with α-GalCer.

Based on the effectiveness of α-GalCer observed using the VSV vector expressing the model OVA antigen, we tested the adenoviral (Ad) vector platform used by our group for HIV vaccine formulations consisting of the first generation Ad vectors (FG-Ad) expressing the HIV envelope and gag antigens (FG-Ad HIV-env and FG-Ad HIV-Gag, respectively) in the presence and absence of α-GalCer adjuvant for intranasal immunization. Serum and vaginal-wash samples from mice in each of the groups collected prior to and at 14 days post-vaccination were analyzed for the presence of antibodies specific to HIV envelope and gag antigens in the vaccine using the standard ELISA protocol (Figure 2A). Significantly higher levels of IgG and IgA antibodies in the serum and vaginal washes, respectively, were detected against both envelope and gag antigens in mice after vaccination in the presence of α-GalCer relative to that in mice vaccinated without the adjuvant, indicating the effectiveness of α-GalCer in enhancing antigen-specific systemic and mucosal antibody responses. Similarly, antigen-specific CTL responses, determined in the splenocytes using the standard chromium release assay, were significantly higher in mice administered the vaccine with α-GalCer relative to those without α-GalCer (Figure 2B). Single cell suspensions collected from different tissues harvested on Day 14 were analyzed for antigen-specific T cells in terms of cytokine production using ELISpot assay (Figure 2C,D). Significantly higher antigen-specific IFN-γ producing CD8 and CD4 T cells were detected in several tissues including lung, lung-draining mediastinal lymph nodes (MdLN) as well as Iliac lymph nodes (ILN), surrogates of genital immune responses (Figure 2C,D). 

Together, these results support α-GalCer to be an effective adjuvant to afford significant improvements for viral vectored vaccines in mice in terms of inducing antigen-specific humoral and cellular immune responses in systemic as well as mucosal tissues.

### 3.2. Induction of NKT Cells Responses in Rhesus Macaques by α-GalCer

**Figure 3:**
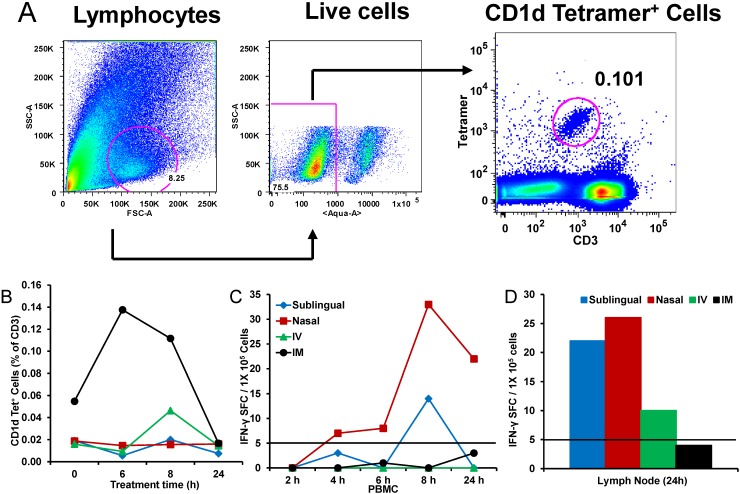
*In vivo* expansion of natural killer T (NKT) cells with α-GalCer delivered by different routes in rhesus macaques: Individual animals were administered α-GalCer as free drug (125 µg) by sublingual, intranasal (IN), intravenous (IV), and intramuscular (IM) routes followed by determining the percentages of NKT cells in the blood by staining with allophycocyanin (APC)-labeled CD1d tetramer pre-loaded with α-GalCer. The gating scheme (**A**) for the analyses of the NKT cells in the peripheral blood mononuclear cells (PBMC) from a representative animal included first selecting the lymphocytes based on forward scatter (FCS) versus side scatter (SSC), and then live lymphocytes were identified based on staining with Aqua Live/Dead reagent (Invitrogen, Carlsbad, CA, USA). The T cells were then positively identified by CD3 expression followed by the detection of the CD1d tetramer^+^ populations within the CD3^+^ T cells. Percentages of CD1d tetramer^+^ NKT cells in PBMC isolated from individual monkeys at 0, 6, 8 and 24 h after delivering α-GalCer by the different routes are shown in Figure 3B. Numbers of IFN-γ producing cells within the blood (**C**) and lymph nodes (**D**) after administering α-GalCer by the different routes in individual monkeys as an indication of activated NKT cells are enumerated by the standard cytokine ELISpot assay. Data are shown as IFN-γ spot forming cells (SFC) per 1 × 10^5^ input cells and antigen specific responses were adjusted to background medium control.

Since immune responses generated by vaccination in mice may not truly predict applicability to humans and also since rhesus macaques represent a suitable nonhuman primate model close to humans, specifically for HIV vaccine approaches, we tested the effectiveness of α-GalCer to induce NKT cells responses in macaques. However, in general the populations of NKT cells in circulation as well as within tissues are relatively low in primates when compared to those in rodents, and systemic administration of α-GalCer as free drug can result in the NKT cell anergy under some conditions [26,27]. Because of this potential problem, most studies rely on administering dendritic cells (DCs) loaded *ex vivo* with α-GalCer to ensure a stimulatory effect [26,28,29]. While DC-mediated vaccination has a role in certain situations, this is not a practical option for gene-based vector-mediated vaccines against infectious diseases like HIV-AIDS that require mass scale immunization campaigns. Given this, the ability to stimulate NKT cells in non-human rhesus macaques was explored. We tested delivering α-GalCer to macaques by four different routes to activate NKT cells: intramuscular (IM), intravenous (IV), intranasal (Nasal), and sublingual (Figure 3). Blood and liver NKT cell populations were analyzed using the CD1d tetramer (Figure 3A). Consistent with previous reports, NKT cell populations were low in the macaques prior to α-GalCer administration. However, IM injection of α-GalCer stimulated robust increases in CD1d-positive NKT cells (Figure 3B). In contrast, α-GalCer administration by the other routes stimulated only weak responses. Since the hall mark for *in vivo* functionality of activated NKT cells is production of high amounts of IL4 and IFN-γ, we tested peripheral blood mononuclear cells (PBMC) isolated at different time points after α-GalCer administration by the four different routes for IFN-γ producing cells and observed the strongest responses from sublingual and IN routes (Figure 3C), which is also reflected in lymph node cells isolated at 24 h post-administration of α-GalCer (Figure 3D). Analyses of serum levels of cytokines at several time points after α-GalCer administration by the four different routes for IL4 and IFN-γ production showed low but detectable levels of IL-4 and IFN-γ after IM and sublingual routes, respectively, relative to other routes (data not shown) [30].

Together, these results show for the first time functional activation of NKT cells in nonhuman primates after directly administering α-GalCer as free reagent without having to pre-load *ex vivo* onto presenting cells such as the DC. 

### 3.3. Intranasal Administration of α-GalCer to Rhesus Macaques Induces NKT Cells Activation and Induces Adaptive Immune Responses to Co-Administered Antigen 

**Figure 4:**
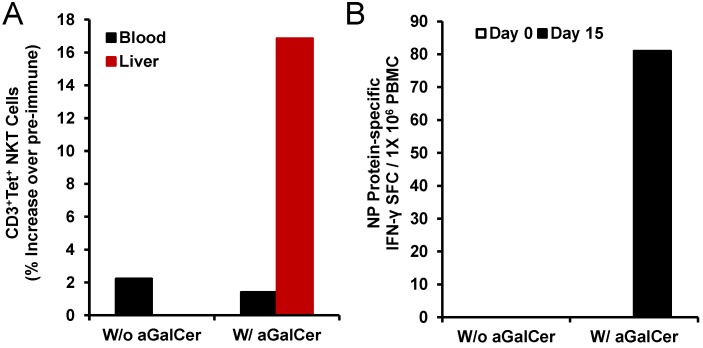
Activation of NKT cell and adaptive immune responses after intranasal delivery of α-GalCer to rhesus macaques: Intranasal immunization with the recombinant nucleoprotein of influenza (NP protein 20 µg) along with, but not without, α-GalCer (125 µg) induced expansion of NKT cells in the blood and liver tissues as determined by flow cytometry staining with APC CD1d tetramer (panel **A**). Data are calculated as percent increase of CD3^+^, CD1d tetramer^+^ NKT cells relative to pre-immune values for one monkey each immunized with the NP protein in the absence or presence of α-GalCer. Adaptive immune responses to the NP protein were determined in terms of IFN-γ producing cells in the PBMC by the cytokine ELISpot assay (panel **B**). Data are shown as IFN-γ spot forming cells (SFC) per 1 × 10^5^ input cells and antigen specific responses were adjusted to background medium control.

To further investigate the *in vivo* adjuvant potential of α-GalCer, we tested in a separate pilot study the immunization of single rhesus monkeys each by the intranasal route with the nucleoprotein (NP) of the influenza virus as a model antigen in the presence and absence of α-GalCer and analyzed for NP-specific T cell and antibody responses (Figure 4). We observed induction of higher numbers of NKT cells in the liver after intranasal immunization with NP in the presence of α-GalCer but not NP alone (Figure 4A). Only in the PBMC isolated from the animal immunized with NP and α-GalCer, we observed induction of NP-specific IFN-γ producing cells (Figure 4B). Immunization with NP along with α-GalCer, but not NP alone resulted in the production of NP-specific IgG antibodies in the blood and IgA antibodies in the bronchial alveolar lavage (BAL) that were above background pre-immune levels (data not shown) [30]. 

These results from the pilot study are suggestive of the *in vivo* effectiveness of α-GalCer as an adjuvant in the nonhuman primate model of rhesus macaques, similar to our data from mice, for improving adaptive immune responses to co-administered antigens.

### 3.4. Enhancement of Systemic and Mucosal Immune Responses after Sublingual Mucosal Vaccination of Rhesus Macaques with Adenoviral Vectored HIV Antigen in the Presence of α-GalCer Adjuvant 

**Figure 5:**
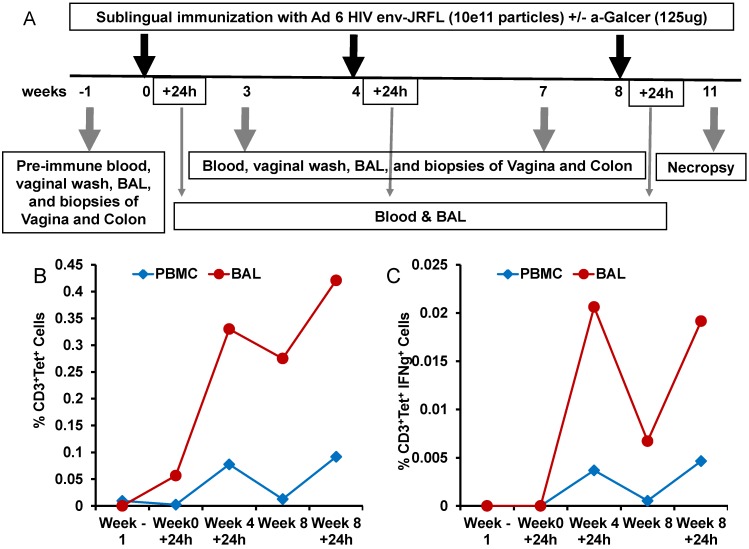
Expansion and activation of NKT cells after sublingual immunization with HD-Ad HIV envelope in the presence of α-GalCer adjuvant: In this pilot study, a single rhesus monkey was immunized by the sublingual route with the HD-Ad vector encoding the HIV-1 envelope protein (10^11^vp) along with α-GalCer (125 µg) as shown in the scheme (**A**). Prior to immunization and at different time points subsequently, blood and bronchial alveolar lavage (BAL) samples were collected and lymphocytes were analyzed by flow cytometry for the percentages of NKT cells in terms of CD3^+^, CD1d tetramer^+^ cells (**B**) and percentages of IFN-γ producing NKT cells in terms CD3^+^, CD1d tetramer^+^, IFN-γ^+^ cells (**C**).

To determine whether the potency of α-GalCer to enhance immune responses to viral vectored antigens observed in mice will also translate into the nonhuman primate model of rhesus macaques, we conducted another pilot study testing α-GalCer as an adjuvant for mucosal sublingual immunization with the HD-Ad vector encoding the HIV envelope antigen (HD-Ad HIV-env). The immunization regimen included sublingual delivery of HD-Ad HIV-env alone or along with α-GalCer to one animal each at weeks 0, 4 and 8 (Figure 5A). The animal immunized with HD-Ad HIV-env in the presence of α-GalCer showed pronounced enhancement of NKT cells numbers as well as IFN-γ production by NKT cells, relative to pre-immune time point, in the bronchia alveolar lavage (BAL) samples and to a lesser extent in the blood (Figure 5B,C). 

**Figure 6:**
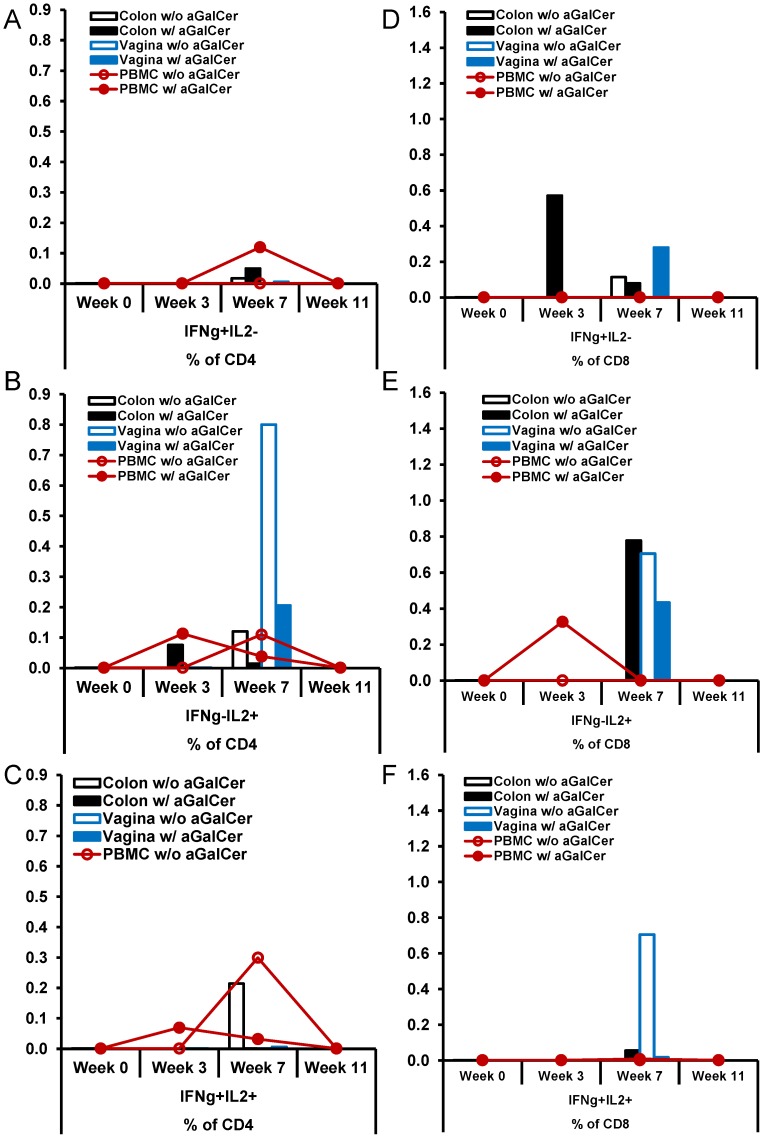
Enhancement of antigen-specific CD4 and CD8 T cells responses after sublingual immunization with HD-Ad HIV envelope in the presence of α-GalCer adjuvant: Single cell suspensions prepared from biopsy tissues of colon and vagina along with PBMC from two rhesus macaques immunized by the sublingual route with the HD-Ad vector encoding the HIV-1 envelope protein (10^11^vp) alone (*n* = 1) or along with α-GalCer (125 µg; *n* = 1), open and closed symbols, respectively, were analyzed by flow cytometry for IFN-γ and/or IL-2 producing CD4 and CD8 T cells. Various tissues at different time points post immunization showing percentages of CD4 T cells (**A**–**C**) and CD8 T cells (**D**–**F**) producing IFN-γ alone (IFN-γ^+^IL2^−^), IL-2 alone (IFN-γ^−^IL2^+^) or both (IFN-γ^+^IL2^+^).

In this pilot study, we also compared the levels of induction and expansion of antigen-specific IL2 and/or IFN-γ producing CD4^+^ T cells (Figure 6A–C) and CD8^+^ T cells (Figure 6D–F) in the blood, colon, and vagina samples at different time points post-immunization. Immunization with HD-Ad HIV-env in the presence of α-GalCer in general yielded higher percentages of CD4^+^ T cells producing IFN-γ in blood, relative to that in the monkey immunized without α-GalCer adjuvant. Similarly, we observed higher percentages of CD8^+^ T cells producing IFN-γ in colon and vagina, and IL-2 producing cells in the blood and colon when HD-Ad HIV-env vaccination was administered with α-GalCer, relative to without α-GalCer.

**Figure 7:**
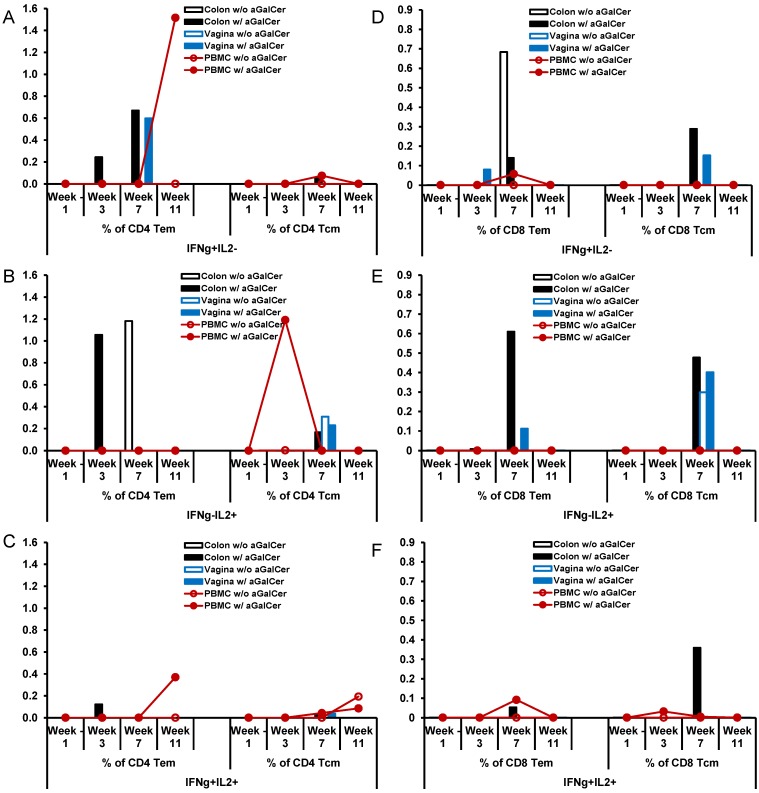
Enhancement of antigen-specific memory subsets of CD4 and CD8 T cells responses after sublingual immunization with HD-Ad HIV envelope in the presence of α-GalCer adjuvant: Single cell suspensions prepared from biopsy tissues of colon and vagina along with PBMC from single rhesus monkey immunized by the sublingual route with the HD-Ad vector encoding the HIV-1 envelope protein (10^11^vp) alone or along with α-GalCer (125 µg), open and closed symbols, respectively, were analyzed by flow cytometry for IFN-γ and/or IL-2 producing effector and central memory subsets (Tem and Tcm, respectively) of CD4 (**A**–**C**) and CD8 (**D**–**F**) T cells. Various tissues at different time points post immunization showing percentages of CD4 T cells and CD8 T cells producing IFN-γ alone (IFN-γ^+^IL2^−^; Figure 7A,D), IL-2 alone (IFN-γ^−^IL2^+^; Figure 7B,E) or both (IFN-γ^+^IL2^+^; Figure 7C,F).

The immunization regimen involving HD-Ad HIV-env immunization in the presence and absence of α-GalCer in the two rhesus macaques also resulted in the establishment of differential levels of antigen-specific IL2 and/or IFN-γ producing effector and central memory subsets of CD4^+^ T cells (Figure 7A–C) and CD8^+^ T cells subsets (Figure 7D–F). Overall, in the animal immunized with HD-Ad HIV-env in the presence of α-GalCer, relative to that in the animal immunized in the absence of α-GalCer, there are more CD4 Tem cells producing IFN-γ alone or both IFN-γ and IL-2 in the blood as well as colon, while single cytokine producing CD4 T_cm_ cells were observed in the blood. In case of CD8 T cell memory subsets, immunization employing the α-GalCer adjuvant resulted in higher percentages of cells producing either one of the cytokines or both cytokines in the colon and vagina tissues. Overall, this data from single monkey pilot studies show a trend towards higher percentages of antigen-specific memory CD4 and CD8 T cells subsets (both effector and central memory cells) in the mucosal tissues when the vaccine was delivered in the presence of α-GalCer when compared to that in the absence of the adjuvant.

**Figure 8:**
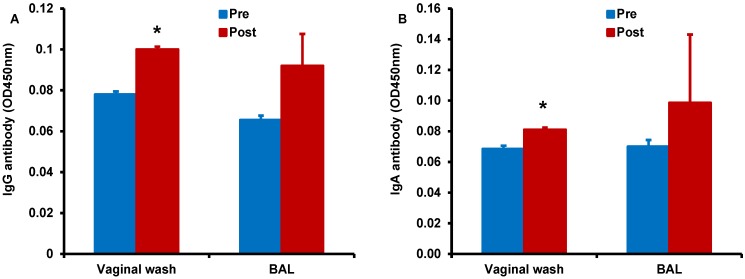
Enhancement of antigen-specific antibody responses after sublingual immunization with HD-Ad HIV envelope in the presence of α-GalCer adjuvant: Vaginal wash and BAL samples collected from monkeys prior to and after immunization (pre and post, respectively) by the sublingual route with the HD-Ad vector encoding the HIV-1 envelope protein (10^11^vp) along with α-GalCer (125 µg) were analyzed by the standard ELISA for IgG (**A**) and IgA (**B**) antibodies specific to the HIV envelope protein gp160. Levels of both types of antibodies were significantly higher in the vaginal wash samples when compared to those in pre-immune samples as determined by the student’s t test (* *p* < 0.05).

Finally, we determined antigen-specific antibody responses in serum, vaginal wash, and BAL samples collected form the monkey immunized by the sublingual route with HD-Ad HIV-env in the presence of α-GalCer adjuvant (Figure 8). We observed higher IgG (Figure 8A) and IgA (Figure 8B) responses in the vaginal wash samples collected after vaccination when compared to pre-immune base level values. Similarly, increases for both IgG and IgA antibodies were observed in the BAL for vaccination including α-GalCer adjuvant relative to pre-immune levels, but these differences did not reach statistical significance. Serum levels of IgG and IgA antibodies were not significantly different between samples pre- and post-immunization (data not shown) [30]. In the monkey immunized with HD-Ad HIV-env in the absence of α-GalCer adjuvant no substantial IgG or IgA responses were observed (data not shown) [30].

Together, these data showing enhanced adaptive responses in terms of T cells and antibodies specific to HIV env in mice as well as rhesus macaques support sublingual immunization employing the α-GalCer adjuvant to be a potentially useful vaccination strategy for enhancing adaptive immune responses.

## 4. Discussion 

The utility of α-GalCer in humans to activate NKT cells and enhance adaptive immune responses has thus far been realized only in clinical trials involving delivery of dendritic cells pre-loaded *ex vivo* with α-GalCer. Data obtained in the present investigation provide the first indication to the advantages of delivering the α-GalCer adjuvant directly as free reagent in the nonhuman primate model of rhesus macaques based on its ability to activate NKT cells *in vivo* and thereby enhance adaptive immune responses.

Vaccination employing antigens expressed from viral vectors, relative to recombinant forms of proteins and subunits as synthetic peptides is advantageous owing to the superiority of the former to deliver antigens to the antigen presenting cells as well as potentially activating innate immune mediators. However, mucosal tissues inherently tolerant to foreign antigens pose unique challenges for priming immune responses in general. Our results from both the mouse and macaque models highlight the effectiveness of α-GalCer adjuvant to enhance adaptive immune responses to vaccine antigens encoded by viral vectors when delivered by the mucosal intranasal and sublingual routes.

While protein and peptide antigens delivered by mucosal routes require co-administration of adjuvant, viral vectors by themselves have been demonstrated to be effective for mucosal immunization. Both VSV and adenoviruses are established vectors for vaccine delivery and their administration by mucosal routes has been explored extensively. Intranasal recombinant VSV and adenovirus expressing hemagglutinin protein have been shown to protect against influenza virus challenge [31,32]. Nevertheless, results from our current studies show that inclusion of the NKT cell agonistic ligand α-GalCer as a mucosal adjuvant with the VSV in mice and adenovirus vector vaccines in mice and macaques resulted in the enhancement of antigen-specific immune responses. Additionally, we and others reported earlier that the concurrent administration of α-GalCer as adjuvant mediates a broader distribution of adaptive immune responses in both systemic and mucosal compartments including respiratory and genital tissues [33] and they can be rapidly recalled following a challenge or re-stimulation [18,34]. Besides the advantage of generating immunity at distant mucosal sites because of the common mucosal immune system, it is important to note that administration of viral vectored vaccines by nasal (mucosal) route also allows for circumventing pre-existing immunity to the viral vectors themselves [35,36]. Furthermore, our earlier reports showed that α-GalCer administration by mucosal routes, as opposed to systemic delivery, avoids anergy of NKT cells, allowing for repeated effective vaccination [19,35,36].

We and others have reported that in mice, NKT cells activated in response to α-GalCer as the stimulatory ligand effectively bridge the innate and adaptive immune responses because of their unique ability to induce rapid and robust cytokine production resulting in secondary activation of a variety of innate and adaptive immune cells. Results from our present investigation showing α-GalCer-mediated activation of NTK cells and consequent production of IL-4 and IFN-γ as well as enhancement in the numbers of cytokine producing cells in the blood and lymph nodes in rhesus macaques further extend the potential utility of α-GalCer as free reagent in the nonhuman primate models. 

The primary aim of this study was to assess the feasibility of α-GalCer as adjuvant to enhance immunogenicity of vaccine candidates in mouse and nonhuman primate models, the latter in small pilot scale studies. There are only a few reports of clinical studies using α-GalCer against malignant diseases. Giaccone *et al*. [37] reported that intravenous administration of α-GalCer (KRN7000) did not result in appreciable changes in the levels of NKT cells but resulted in measurable increases in the serum levels of IFN-γ, IL-12, GMCSF, and TNF-a, serving as surrogate for activation of NKT cells and potential subsequent activation of DC. Similarly, Taniguchi *et al.* [38] also observed a rapid but transient disappearance of Va24 NKT cells from the peripheral blood. While our studies also showed very low levels of circulating NKT cells in rhesus macaques, we observed that α-GalCer administration, specifically by the intranasal and sublingual routes showed increasing trends for the number of CD1d tetramer^+^ NKT cells in blood and cytokine (IFN-γ) producing cells in the blood and lymph nodes. In this regard, we reported earlier that mucosal intranasal delivery of α-GalCer but not after systemic intravenous route enabled presentation of α-GalCer by DC and increased levels of NKT cells and subsequent increases in adaptive immune responses to co-administered antigens in mice, suggesting the importance of mucosal α-GalCer delivery [19]. 

## 5. Conclusions

In conclusion the present investigation demonstrates the effectiveness of mucosal intranasal immunization with α-GalCer at enhancing adaptive T cell and antibody responses in the rhesus macaque model with the NP protein of influenza and in the murine model with recombinant viral vectored vaccine antigens. Although with rhesus macaque model the data presented is obtained from small scale pilot studies and it is necessary to conduct experiments employing bigger cohorts of animals to realize the complete potential of α-GalCer as a mucosal adjuvant, the current study indicates that this is an easier and safer vaccination strategy for improvement of antigen-specific immunity.
